# Supplementary material for: Observation of phonon Stark effect
Source: Nat Commun. 2024 May 29;15:4586. doi: 10.1038/s41467-024-48992-w (PMC11137145; doi:10.1038/s41467-024-48992-w)
Supplement: Supplementary file 1 — Supplementary Information for Observation of phonon Stark effect [file 41467_2024_48992_MOESM1_ESM.pdf]

Supplementary Information for

Observation of phonon Stark effect

Zhiheng Huang[1,2†], Yunfei Bai[1,2†], Yanchong Zhao[1,2], Le Liu[1,2], Xuan Zhao[1,2], Jiangbin Wu[3], Kenji Watanabe[4], Takashi Taniguchi[5], Wei Yang[1,2], Dongxia Shi[1,2], Yang Xu[1,2], Tiantian Zhang[6], Qingming Zhang[1,2,7], Ping-Heng Tan[3], Zhipei Sun[8], Sheng Meng[1,2,9], Yaxian Wang[1]*, Luojun Du[1,2]* and Guangyu Zhang[1,2,9]*

[1]Beijing National Laboratory for Condensed Matter Physics; Key Laboratory for Nanoscale Physics and Devices, Institute of Physics, Chinese Academy of Sciences, Beijing, 100190, China

[2]School of Physical Sciences, University of Chinese Academy of Sciences, Beijing 100190, China

[3]State Key Laboratory of Superlattices and Microstructures, Institute of Semiconductors, Chinese Academy of Sciences, Beijing, China

[4]Research Center for Functional Materials, National Institute for Materials Science, 1-1 Namiki, Tsukuba 305-0044, Japan

[5]International Center for Materials Nanoarchitectonics, National Institute for Materials Science, 1-1 Namiki, Tsukuba 305-0044, Japan

[6]CAS Key Laboratory of Theoretical Physics, Institute of Theoretical Physics, Chinese Academy of Sciences, Beijing 100190, China

[7]School of Physical Science and Technology, Lanzhou University, Lanzhou 730000, China

[8]QTF Centre of Excellence, Department of Electronics and Nanoengineering, Aalto University, Tietotie 3, FI-02150 Espoo, Finland

[9]Songshan Lake Materials Laboratory, Dongguan, Guangdong Province 523808, China

[†]These authors contributed equally to this work: Zhiheng Huang, Yunfei Bai

*Corresponding authors. Email: luojun.du@iphy.ac.cn; yaxianw@iphy.ac.cn; gyzhang@iphy.ac.cn

Supplementary Note 1:

Supplementary Figure 1 shows the optic images of all three measured high-quality *h*-BN encapsulated dual-gate bilayer 2*H* MoS_2 devices (labelled as H1-H3) with the same structure schematically shown in Fig. 1a in the main text. The top graphene, top *h*-BN, ground graphene, bilayer MoS_2, bottom *h*-BN and bottom graphene are respectively outlined by the green, pink, navy, red and brown dashed lines.

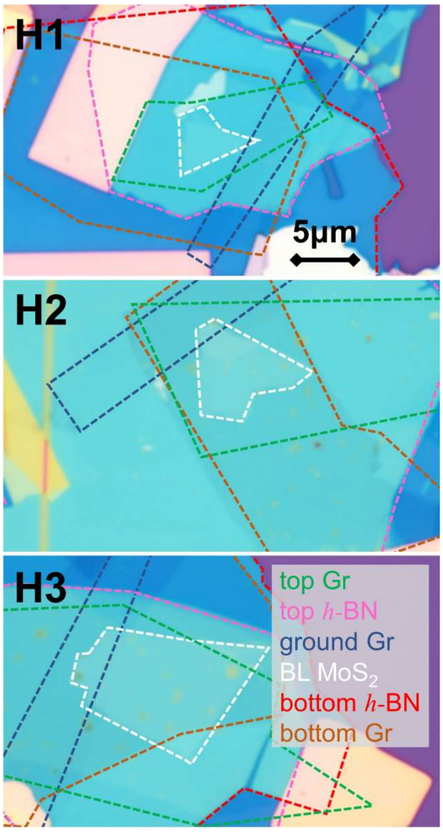

Supplementary Figure 1: Optic microscope images of the fabricated high-quality *h*-BN encapsulated dual-gate bilayer 2*H* MoS_2 devices. Gr: graphene; BL MoS_2: bilayer MoS_2.

Supplementary Note 2:

Based on previous results [1,2], the non-vanishing interlayer hopping $2t_\perp$ (0.086 eV) for holes leads to the hybridization of the valence band states with the same spin from upper (L1) and lower layers (L2) in bilayer 2*H* MoS_2. Here we take the IX species schematically shown in Fig. 1b as an example. The interlayer hole hybridization can be described by a two-level Hamiltonian model: $\begin{pmatrix} \Delta & t_\perp \\ t_\perp & -\Delta \end{pmatrix}$, and thus two hybridized eigenstates with energy separation of $2\sqrt{\Delta^2 + t_\perp^2}$ emerge [3]: $|\psi_+\rangle = \frac{1}{\sqrt{2}}(\cos\alpha \cdot |\psi_1\rangle + \sin\alpha \cdot |\psi_2\rangle)$ and $|\psi_-\rangle = \frac{1}{\sqrt{2}}(\cos\alpha \cdot |\psi_1\rangle - \sin\alpha \cdot |\psi_2\rangle)$. Here, the basis $|\psi_1\rangle/|\psi_2\rangle$ is the wave-function of decoupled upper/lower layer, 2Δ (0.147 eV) denotes the spin-orbit splitting in the valence band of bilayer 2*H* MoS_2, and $\cos 2\alpha = \frac{\Delta}{\sqrt{\Delta^2 + t_\perp^2}}$ (Supplementary Figure 2) [1-3]. According to the hybridized hole wavefunctions, the in-built electric dipole moment for IXs can be calculated as $edcos^2\alpha = 0.606\ e \cdot nm$. Here $d = 0.65\ nm$ denotes the interlayer distance of bilayer 2*H* MoS_2.

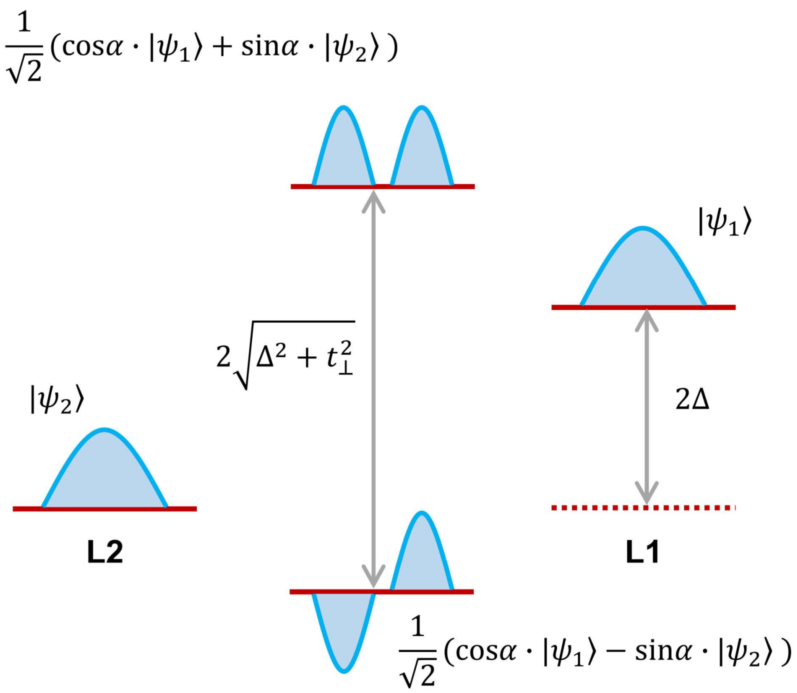

Supplementary Figure 2: Schematic of the interlayer hole hybridization in bilayer 2*H* MoS_2.

Supplementary Note 3:

Supplementary Fig. 3 shows the contour plot of raw Raman spectra of device H1 against F_z. As IXs are tuned to converge the LA phonon line around 230 cm^{-1}, a remarkable phonon mode (labelled as SP) emerges and redshifts with further rising F_z, indicating a linear phonon Stark effect. To clarify this fine signature, we show the contour plot of the corresponding $\partial I/\partial\omega$ in Fig. 2a in the main context.

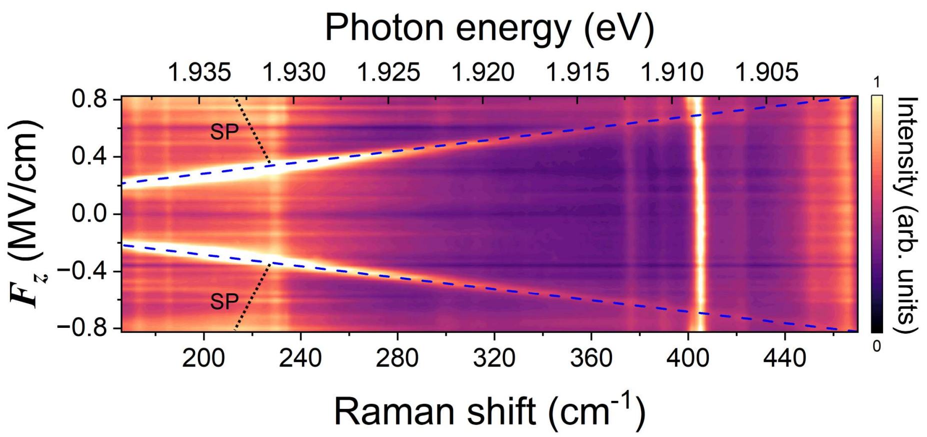

Supplementary Figure 3: Contour plot of the Raman spectra of device H1 as a function of Raman shift (bottom axis) and F_z (left axis). The Stark shift of SP mode is denoted by black dotted lines. IX_1 and IX_2 are respectively denoted by green and blue dotted lines.

Supplementary Note 4:

Supplementary Figure 4 presents the contour plots of raw Raman data (Supplementary Fig. 4a) and corresponding $\partial I/\partial \omega$ (Supplementary Fig. 4b) of device H3 as a function of Raman shift (bottom axis) and electric field F_z (left axis). The linear phonon Stark shift can be clearly observed, consistent with the results of devices H1 and H2.

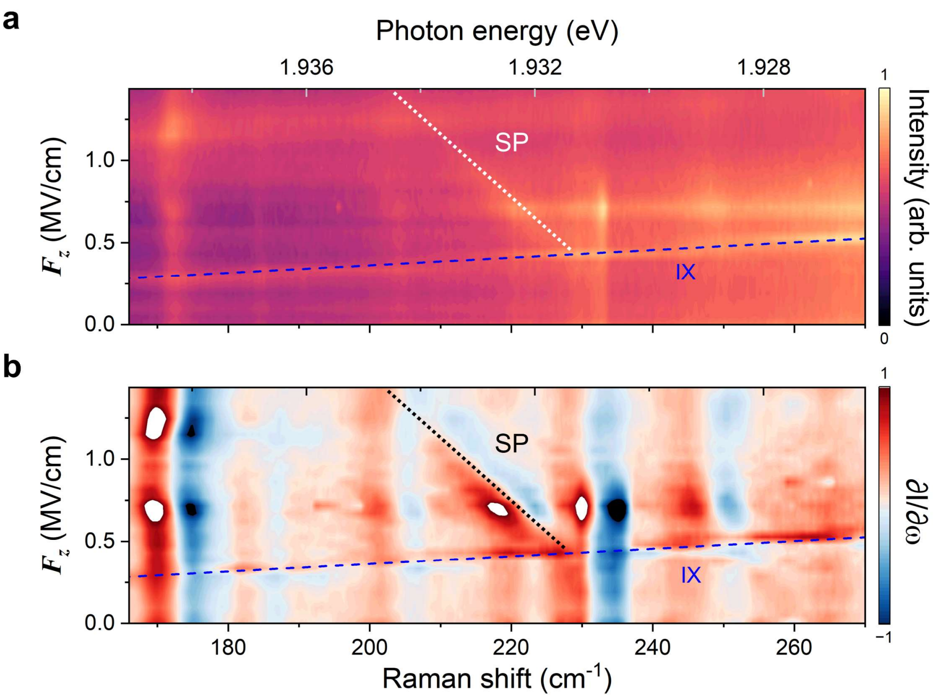

Supplementary Figure 4: a, Contour plots of the Raman spectra respectively ranging from 166 to 270 cm^{-1} of device H3 as a function of Raman shift (bottom axis) and electric field F_z (left axis). **b,** First-order derivative of **a**. Navy dashed lines in **a** and **b** represent IX that are fitted from extracted energies as a function of F_z in device H3. White dotted line in **a** and black dotted line in **b** represent the linear phonon Stark effect.

Supplementary Note 5:

Supplementary Figure 5a shows the fitted emission intensities of LA phonon as a function of electric field. Clearly, LA phonon is activated when IXs are electrically tuned across its emission line. After activation, the intensity of LA phonon first increases and then decreases with the electric field. Since we cannot determine the LA phonon intensity under zero electric field, we define the electro-phonon modulation depth as $\rho = \frac{I - I_m}{I_m}$, where I (I_m) represents the phonon emission intensity at finite (maximum) electric field. Supplementary Figure 5b presents the electro-phonon modulation depth ρ of LA phonon against electric fields. The maximum modulation depth ρ of LA phonon mode can reach ~ 800%. We remark that the modulation depth ρ of LA phonon is much larger than that of A_{1g} phonon, and only slightly smaller than that of A_{2u} phonon, as shown in Fig.4b in the main context.

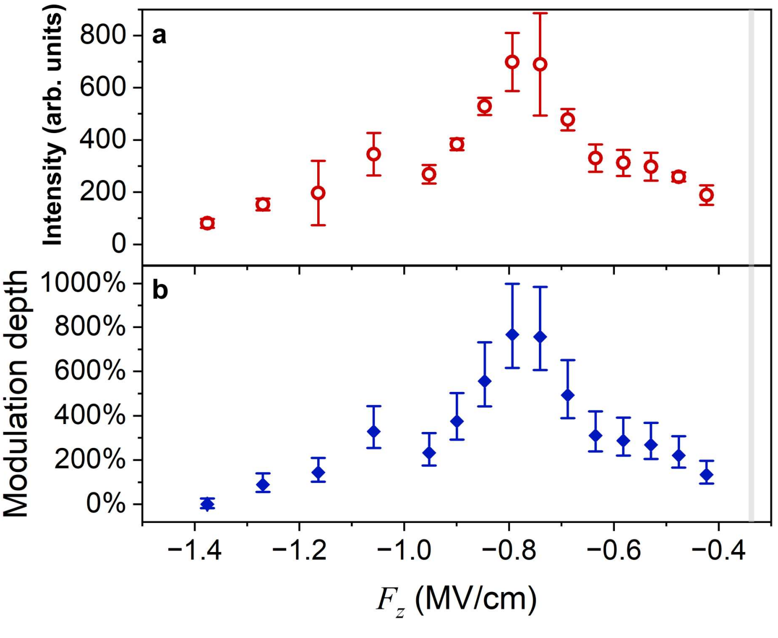

Supplementary Figure 5: a, The fitted emission intensities of LA phonon as a function of electric field. **b,** The electro-phonon modulation depth ρ of LA phonon against electric fields.

Supplementary Note 6:

We have extracted the full width at half maximum (FWHM) of the IXs by fitting for devices H1 (black circles), H2 (red circles) and H3 (navy circles), as shown in Supplementary Fig. 6. For all the three devices, the FWHM of the IXs is very narrow and only ~3.5 meV on average.

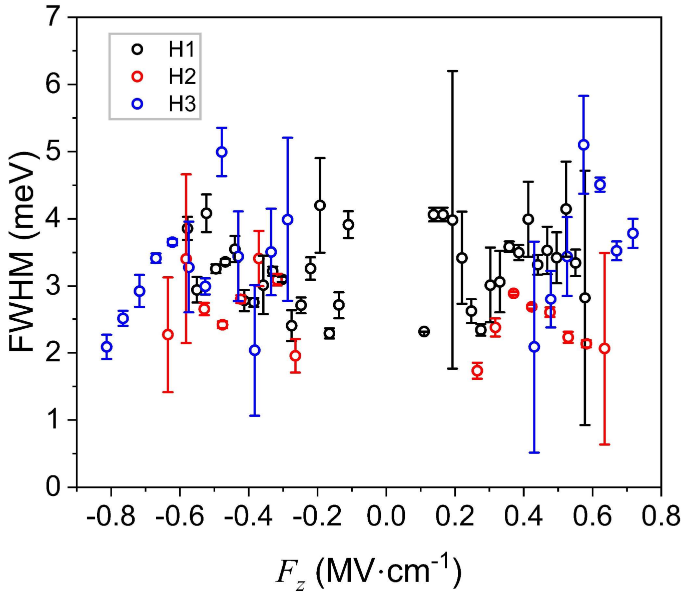

Supplementary Figure 6: Fitted FWHM of IXs in devices H1 (black circles), H2 (red circles) and H3 (navy circles).

Supplementary Note 7:

For a Stokes Raman scattering process (left panel of Supplementary Fig. 7), an electron in ground state is first excited by an incident photon to an intermediate state E_1 with an energy equal to that of the excitation photon E_{ex}, and then scattered to another intermediate state E_2 by emitting a phonon with energy $h\omega_{ph}$ (i.e., the energy of intermediate state E_2 is $E_{ex} - h\omega_{ph}$). The electron in the intermediate state E_2 recombines with the hole in the ground state to emit a photon with energy of $E_{ex} - h\omega_{ph}$, which is detected by the Raman instrument. Employing different excitation phonons (i.e., 633 nm excitation or 532 nm excitation), the corresponding energies of the intermediate states E_1 and E_2 are completely different. Only when a suitable excitation laser is used, and therefore the corresponding intermediate state E_1 or E_2 is close to the IX state, can the IX state involve in the Raman scattering process and thus affect the phonon state via exciton-phonon coupling.

In our case with a 633 nm laser excitation, the energy of intermediate state E_1^{633} is ~1.959 eV, and the energy of intermediate state E_2^{633} is ~1.93 eV given the LA phonon energy is ~0.029 eV. Considering that the IX has the energy of ~1.951 eV at zero electric field which is only ~0.021 eV above intermediate state E_2^{633} and can be easily tuned to resonate with it by electric fields, the IX state can participate in the Raman scattering process and thus renormalise the energies or emission intensities of phonon state via strong exciton-phonon coupling. It is noteworthy that previous work has widely elucidated that the enhancement of phonon emission intensity or activation of Raman silent phonon due to exciton-phonon coupling can only occur under resonant excitation[4-9].

By contrast, for the case of a 532 nm laser excitation, the energy of intermediate state E_1^{532} is ~2.33 eV, and the energy of intermediate state E_2^{532} is ~2.301 eV given the LA phonon energy is ~0.029 eV. Because the IX state is well below (more than 350 meV) the intermediate states E_2^{532} and E_2^{532}, it does not participate in the Raman scattering process and thus we cannot observe the effect of exciton-phonon coupling on the phonon states.

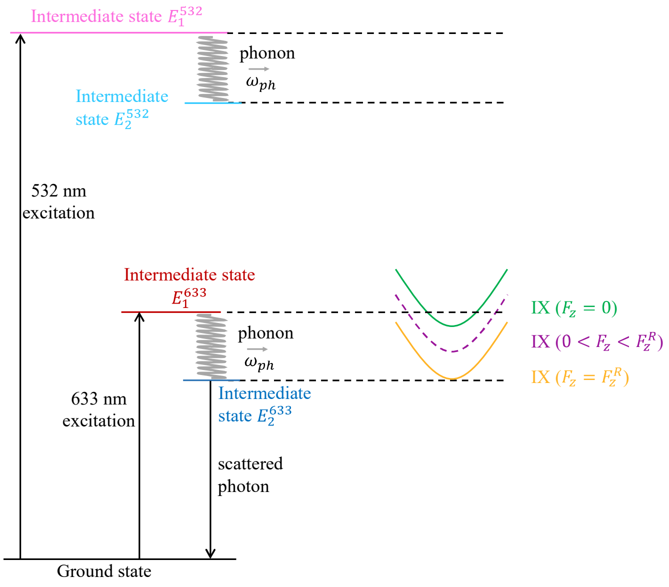

Supplementary Figure 7: Left panel: schematic of a Stokes Raman scattering process under different excitation photons. Right panel: Energy distributions of IX at three selected F_z.

Supplementary Note 8:

Supplementary Fig. 8a (Fig. 8b) shows the emission spectral lines of IXs (LA phonon). Both of them are symmetric, and can be perfectly fitted by Lorentz function. This is in stark contrast to the optical Fano effect which typically gives asymmetric Fano resonance curves. In addition, we highlight that the blue shift of the IX lines in cm^{-1} unit under applied electric field F_z is not due to the optical Fano effect, but because IXs have an out-of-plane static electric dipole and thus show linear Stark shift under F_z as addressed in the main context. Moreover, if the observed phenomena are due to the optical Fano effect, a change of the slope of IXs should be expected when IXs are tuned across the phonon line. This is not the case of our work where the slope of IXs has remained constant (Figs. 1d, 1e, 2a-2c in the main text), ruling out the optical Fano effect.

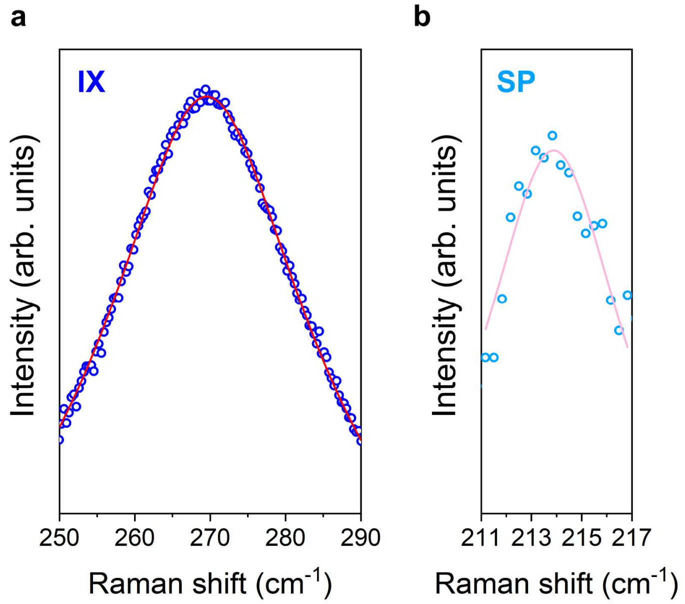

Supplementary Figure 8: The emission spectral lines of IXs (a) and LA phonon (b) for device H2.

Supplementary Note 9:

To visualize the intensity change of $A_{2u}(\Gamma)$ mode mediated by IXs, we first show the typical raw Raman spectra of device H2 before [F_z = 0 MV/cm (blue circles in Supplementary Fig. 9a)] and after [F_z = -0.7937 MV/cm (green circles in Supplementary Fig. 9a)] IXs are tuned across 406 cm^{-1}. By Voigt-fitting, the vast intensity modulation of infrared-active $A_{2u}(\Gamma)$ mode (pink peaks in Supplementary Fig. 9a) can be clearly observed while the intensity modulation of Raman-active $A_{1g}(\Gamma)$ mode (violet peaks in Supplementary Fig. 9a) is much smaller. Note that a slight blueshift of infrared-active $A_{2u}(\Gamma)$ mode and thus a fine decline of Davydov splitting can be observed, as thoroughly discussed in the main context. We also present Raman spectra at diverse F_z to confirm the intensity modulation in Supplementary Fig. 9b.

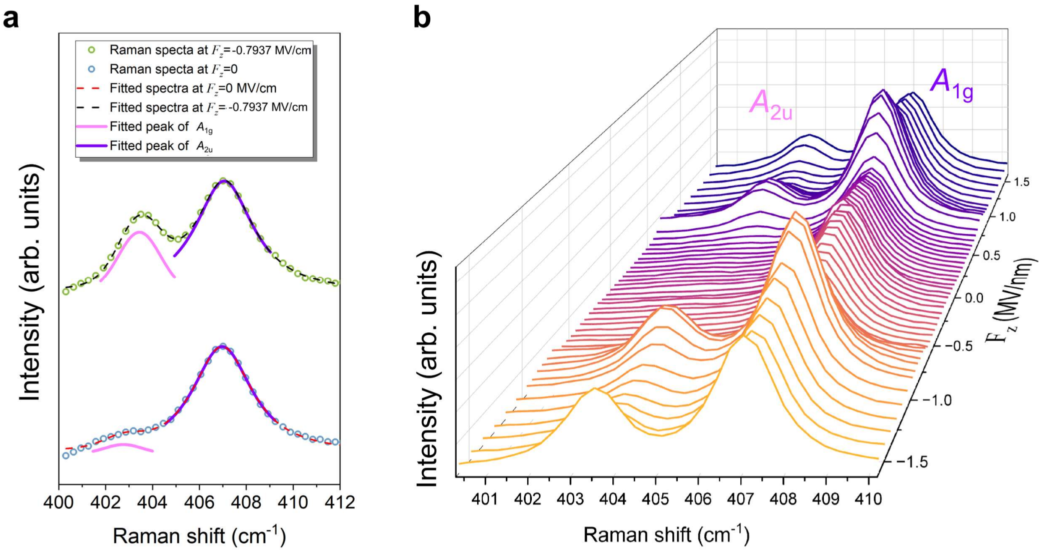

Supplementary Figure 9: a, Raw Raman spectra of H2 at F_z = 0 MV/cm (blue circles) and F_z = -0.7937 MV/cm (green circles). The Voigt-fitted A_{2u} and A_{1g} phonon peaks are respectively shown in pink and violet. The whole fitted curve at F_z = 0 MV/cm (F_z = -0.7937 MV/cm) are present as red (black) dashed line. Vertical offset is set for clarity. **b,** Line-cuts of Fig. 4a as a function of Raman shift at diverse F_z.

Supplementary Note 10:

h-BN encapsulated dual-gate bilayer 3R MoS_2 device is fabricated using the same process as h-BN encapsulated dual-gate bilayer 2H MoS_2 device mentioned in the main context. The optical microscope image of the fabricated h-BN encapsulated dual-gate bilayer 3R MoS_2 device is shown in Supplementary Fig. **10**a. Note that the bilayer 3R MoS_2 is confirmed by room-temperature SHG measurements excited by 780 nm laser, which exhibits ~ 4 times the SHG emission intensity of monolayer (Supplementary Fig. **10**b) [10]. The colour contours of F_z-dependent Raman spectra and their corresponding first derivative are respectively shown in Supplementary Figs. **10**c and **10**d. No noticeable signature of phonon Stark effect or intensity modulations is observed, indicating a vital role of IXs in the phonon Stark effect and electro-phonon modulation of bilayer 2H MoS_2 discussed in main context.

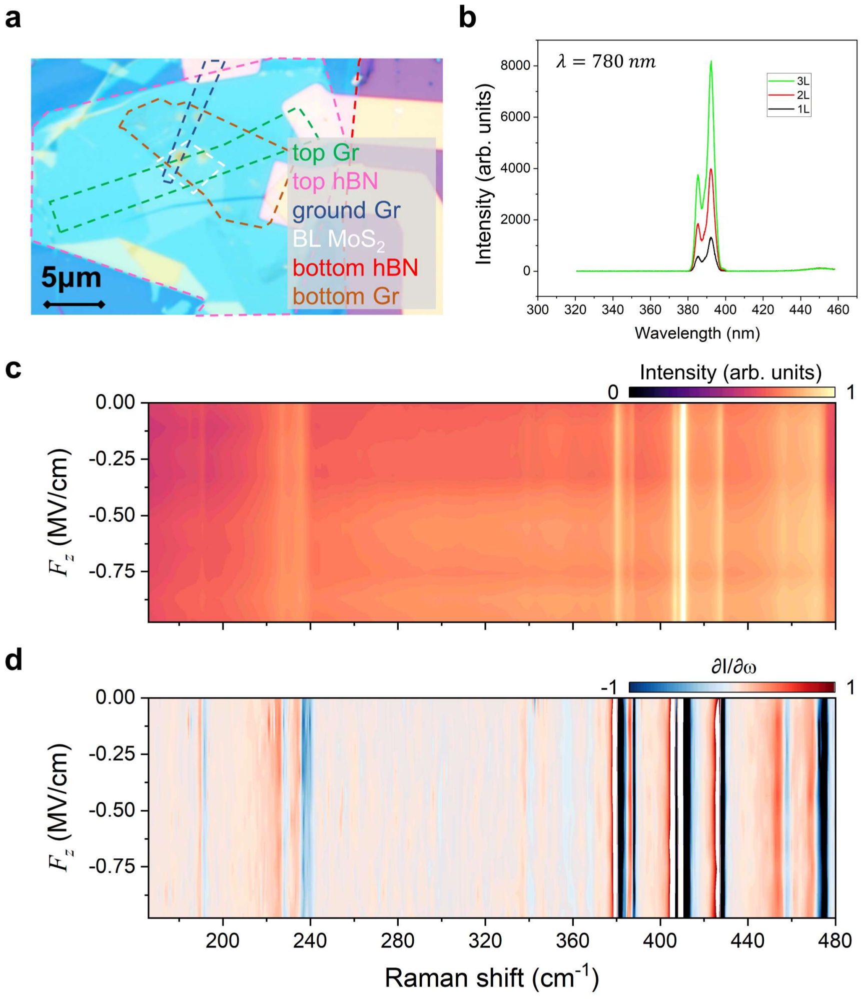

Supplementary Figure 10: a, Optic microscope images of the fabricated high-quality h-BN encapsulated dual-gate bilayer 3R MoS_2 device. Gr: graphene; BL MoS_2: bilayer MoS_2. **b,** SHG spectra of monolayer (black curve), bilayer (red curve) and trilayer 3R MoS_2 (green curve) recorded by 780 nm laser. **c, d,** Contour plot of the Raman spectra (**c**) and the corresponding first-derivative (**d**) of a typical bilayer 3R MoS_2 device as a function of Raman shift (bottom axis) and electric field F_z (left axis).

Supplementary Note 11:

Although stress or strain may also cause modulation of phonon states, we believe that this is not the case of our results. First, apart from the LA(M) phonon, other phonon modes, for example E_{2g} at ~ 385 cm^{-1} and A_{1g} at ~ 405 cm^{-1} that are sensitive to stress or strain, do not show energy shift with electric field. Second, as shown in Note 10, no apparent phonon Stark effect or intensity modulation is observed under the same experimental conditions for bilayer 3*R*-MoS_2, which exhibits similar electronic structure and phonon dispersion to the bilayer 2*H* counterpart, but lacks the IXs. This largely rules out the possibility of stress or strain.

To further confirm that stress or strain does not underlie the observed phonon Stark effect, we performed the electric field-dependent Raman spectra of bilayer 2*H*-MoS_2 under 532 nm excitation (Supplementary Fig. 11), bilayer 2*H*-$MoSe_2$ (Supplementary Fig. 12) and bilayer 2*H*-WS_2 (Supplementary Fig. 13) under 633 nm excitation. For these off-resonance excitation cases (i.e., phonon-IX coupling is absent), no noticeable signature of phonon Stark effect or intensity modulation is observed. This rules out the possibility of the strain mechanism, and confirms that the observed phonon Stark effect in bilayer 2*H*-MoS_2 results from the strong coupling between phonons and IXs.

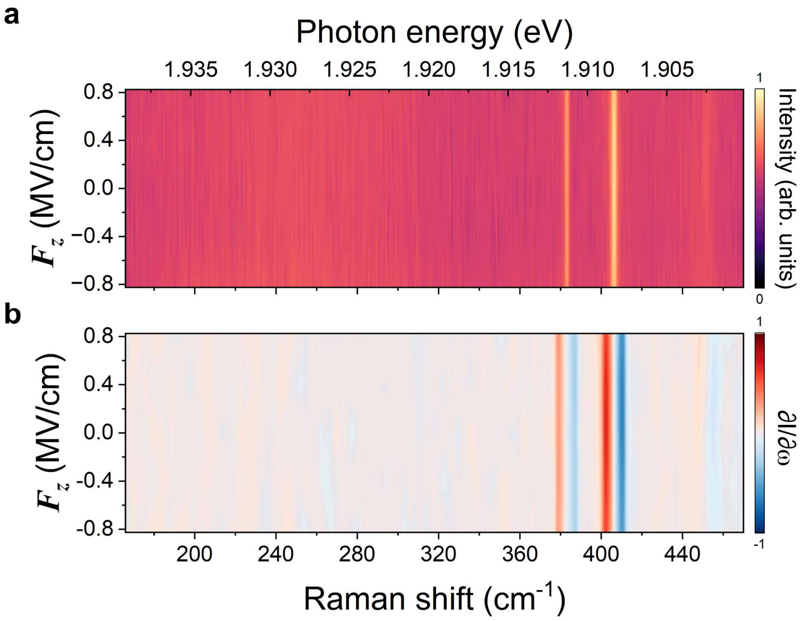

Supplementary Figure 11: a, Raman spectra of bilayer 2*H*-MoS_2 (device H1) under 532 nm excitation as a function of electric field. **b,** First derivative of **a**.

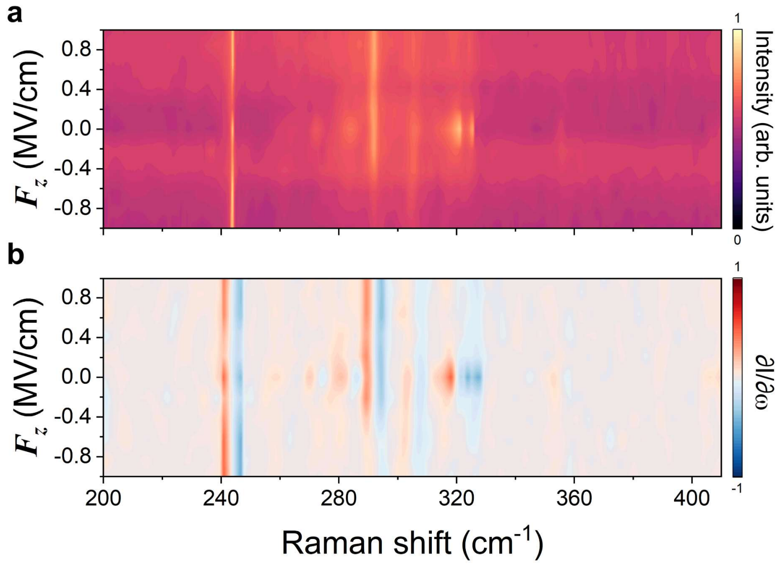

Supplementary Figure 12: a, Raman spectra of a bilayer 2*H*-$MoSe_2$ device under 633 nm excitation as a function of electric field. **b,** First derivative of **a**.

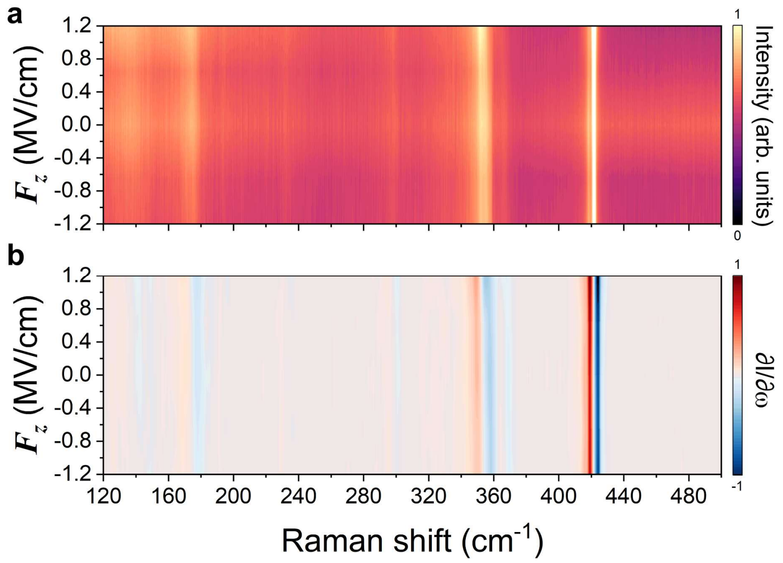

Supplementary Figure 13: a, Raman spectra of a bilayer 2*H*-WS_2 device under 633 nm excitation as a function of electric field. **b,** First derivative of **a**.

Supplementary Note 12:

Supplementary Figure 14a shows the PL spectra of device H3 excited by 532 nm (black curve) and 633 nm lasers (red curve) under zero electric field. The A exciton of bilayer MoS_2 can be clearly distinguished around ~1.923 eV under an off-resonance 532 nm excitation. By contrast, the bilayer MoS_2 A exciton, although can be seen, is largely obscured by the strong signal of phonons under an on-resonance 633 nm excitation. Supplementary Figure 14b shows the PL spectra at selected electric fields under 633nm excitation. Obviously, when the IX is tuned close to bilayer MoS_2 A exciton, its intensity is quite strong and would also largely obscure the A exciton.

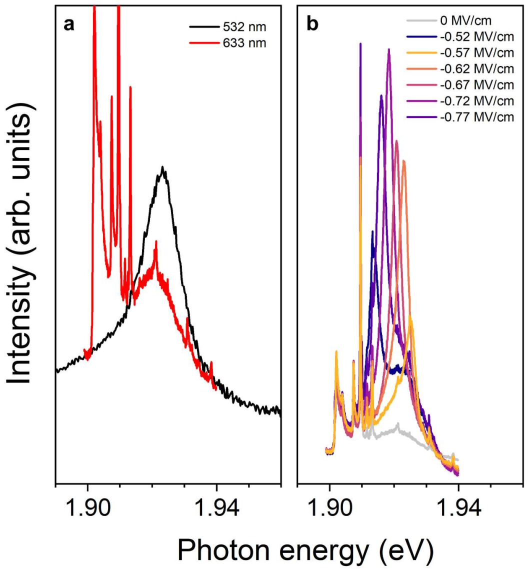

Supplementary Figure 14: a, PL spectra of device under 532 nm (black) and 633nm (red) excitation at zero electric field. **b,** PL spectra at selected electric fields under 633nm excitation.

Supplementary Note 13: Discussion on optical Fano effect

We highlight that although the optical Fano effect between the Raman scattering and a continuum background has the potential to cause variations in the measured phonon energy, we believe that our observed phonon Stark effect does not originate from optical Fano effect. Here we show more details.

1) For Fano shape profile, the spectral shape can be described by the following equation[11-14]:

$$I(\omega) = I_0 \frac{[1 + (\omega - \omega_0)/(q\gamma)]^2}{1 + [(\omega - \omega_0)/\gamma]^2} \qquad (1)$$

Here, I_0 (γ) is the intensity (FWHM) of uncoupled discrete state. $\frac{1}{q}$ denotes the Fano coupling strength, or more specifically the overlap, between the continuum and the discrete state. In the light of the Equation (1), we can derive the energy renormalisation of the discrete state[12,13]:

$$\Delta\omega = \omega - \omega_0 = \frac{\gamma}{q} \qquad (2)$$

As q approaches 0, a larger spectral overlap and thus a stronger Fano coupling strength is expected, giving rise to a larger energy renormalisation $\Delta\omega$.

Thus, if the observed phonon Stark effect originates from optical Fano effect, the maximum phonon energy renormalisation should take place when the IX emission line is tuned to just intersect the LA phonon line (i.e., the positions marked by solid cyan circles in Supplementary Fig. 15) where the Fano coupling strength is strongest. By contrast, our results show that when the IX emission line is tuned to resonate with LA phonon line, the phonon mode just starts to redshift with the applied electric field, while the phonon energy renormalisation is negligible (Supplementary Fig. 15). The apparent contradiction between our measured results and what is expected from optical Fano effect strongly rules out the optical Fano effect as a possible mechanism for the phonon Stark effect we observed.

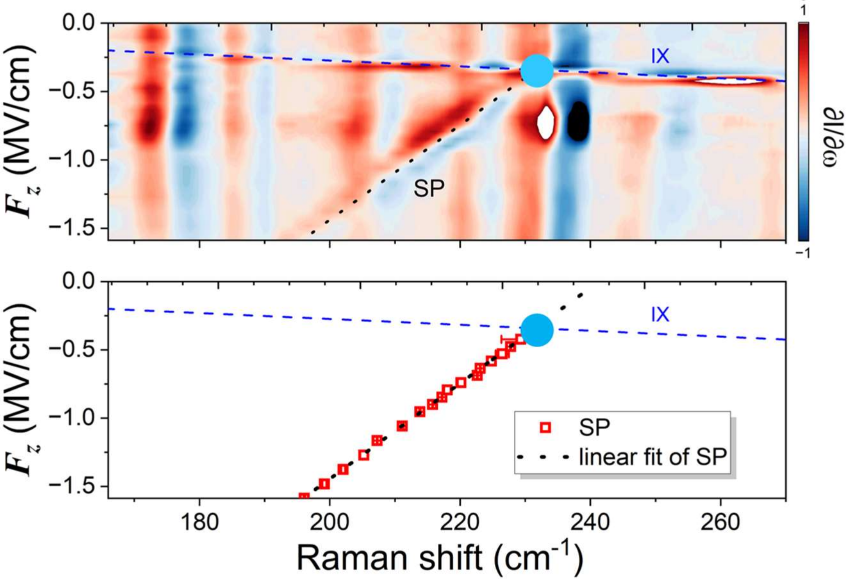

Supplementary Figure 15 (adopted from Fig. 2 in the main text): Upper: Contour plot of the first-order derivative of Raman intensity as a function of phonon energy (bottom axis) and electric field F_z (left axis). Lower: Extracted phonon energy as a function of F_z. The solid cyan circles mark the positions where IX is tuned to intersect the LA phonon line.

2) In the light of the Equation (1), we can derive the FWHM of the discrete state[12,13]:

$$\mathrm{FWHM} = \frac{\gamma(q^2+1)}{|q^2-1|} \qquad (3)$$

Clearly, the FWHM of the discrete state is dependent on the q. Supplementary Fig. 16 shows the fitted FWHM of the SP phonon state against the electric field F_z. The FWHM and thus q basically don't change with the electric field. Together with Equation (2), we can deduce that the phonon energy does not renormalise with the electric field. This is in stark contrast to the optical Fano effect and therefore rules out the optical Fano effect as a possible mechanism for the phonon Stark effect we observed.

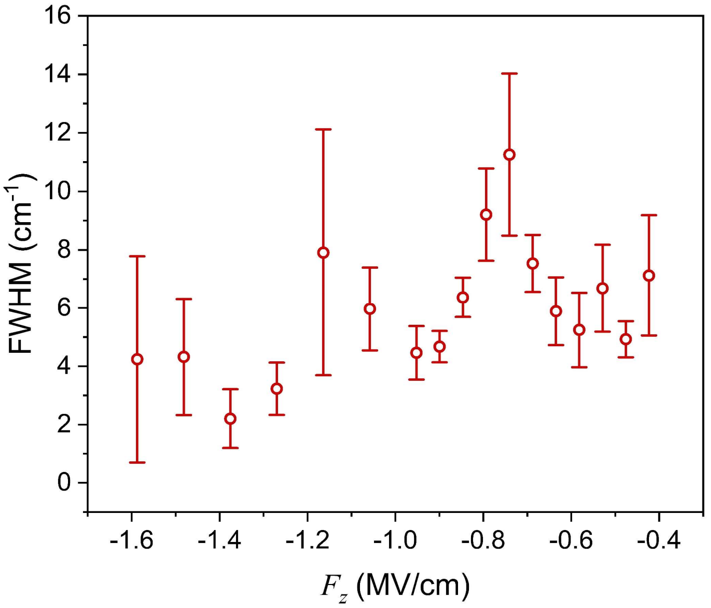

Supplementary Figure 16: The FWHM of SP phonon state as a function of electric field F_z in device H2.

3) If the observed phonon Stark effect originates from optical Fano effect, an almost anti-crossing looking line shape should emerge when IX resonance matches the Raman resonance. However, we do not observe anti-crossing signature for all the devices we have measured. This also effectively excludes the possibility that the optical Fano effect underlies the phonon Stark effect we observed.

4) We highlight that the linear shift of phonon energy with the applied electric fields does not require the spectral overlap between IX and phonon states. Supplementary Fig. 17 shows the Raman spectra of device H2 at three selected electric fields, i.e., F_z=-0.423, -0.476 and -0.529 MV/cm. Clearly, although there is no noticeable spectral overlap between IX and phonon states, we can unequivocally distinguish the phonon Stark effect. This largely rules out the spectra overlap and therefore optical Fano effect as a possible mechanism for the phonon Stark effect we observed.

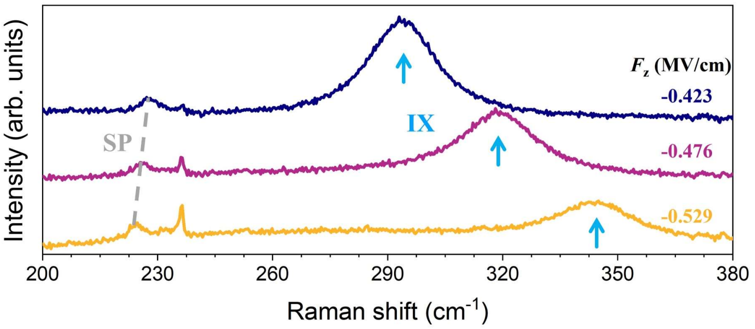

Supplementary Figure 17: On-resonant Raman spectra of device H2 at three selected F_z. Signals of SP states (IXs) are marked by the grey dashed line (lime blue arrows). Note that each spectrum is acquired by subtracting the raw data at $F_z = 0$ to eliminate the influence of A exciton. There is no notable overlap between SP and IX but SP still redshifts linearly with increasing F_z, ruling out spectra overlap as a possible mechanism of phonon Stark effect.

Supplementary Note 14: Computational details

All optical excitonic transitions are calculated using the state-of-art *GW-BSE* approach (*GW*, one-body Green's function *G* and the dynamically screened Coulomb interaction *W*; *BSE*, Bethe-Salpeter equation, which is available in multiple *ab initio* codes[15,16] and can give the exciton wavefunction at finite momentum[17]. Following the implementation in Yambo code, the Bethe-Selpeter equation can be reduced to an eigenvalue problem of the Hamiltonian *H*, i.e. the H_{BSE} in our implementation, as

$$H_{\substack{nn'\mathbf{k}\\ mm\mathbf{k}'}} = (\varepsilon_{n\mathbf{k}} - \varepsilon_{n'\mathbf{k}})\delta_{nm}\delta_{n'm'}\delta_{\mathbf{kk}'} + (f_{n'\mathbf{k}} - f_{n\mathbf{k}})\left[2\bar{V}_{\substack{nn'\mathbf{k}\\ mm'\mathbf{k}}} - W_{\substack{nn'\mathbf{k}\\ mm'\mathbf{k}}}\right]. \quad (2)$$

Here both W and $\bar{V}$ are integrals of the Bloch functions

$$W_{\substack{nn'\mathbf{k}\\ ss'\mathbf{k_1}}} = \frac{1}{\Omega N_q}\sum_{\mathbf{GG}'}\rho_{ns}(\mathbf{k}, \mathbf{q} = \mathbf{k} - \mathbf{k_1}, \mathbf{G})\rho^*_{n's'}(\mathbf{k_1}, \mathbf{q} = \mathbf{k} - \mathbf{k_1}, \mathbf{G}') \times \epsilon^{-1}_{\mathbf{GG}'}v(\mathbf{q} + \mathbf{G}'),$$

$$\bar{V}_{\substack{nn'\mathbf{k}\\ ss'\mathbf{k_1}}} = \frac{1}{\Omega N_q}\sum_{\mathbf{G}\neq 0}\rho_{nn'}(\mathbf{k}, \mathbf{q} = \mathbf{0}, \mathbf{G})\rho^*_{ss'}(\mathbf{k_1}, \mathbf{q} = \mathbf{0}, \mathbf{G})v(\mathbf{G}), \quad (3)$$

where N_q the number of points in the Brillouin zone (BZ) sampling, Ω the unit cell volume, and $v(\mathbf{q} + \mathbf{G}) = \frac{4\pi}{|\mathbf{q}+\mathbf{G}|^2}$. $\epsilon^{-1}_{GG'}$ is the random-phase approximation (RPA) dielectric function. One can see that the electron-electron scattering term (*W*) and the exchange interaction ($\bar{V}$) now has a combination of conduction and valence band index as ($|v\mathbf{k}\rangle$ the single particle level) $\rho_{nm}(\mathbf{k}, \mathbf{q}, \mathbf{G}) = \langle n\mathbf{k}|e^{i(\mathbf{q}+\mathbf{G})\mathbf{r}}|m\mathbf{k} - \mathbf{q}\rangle$. We include four valance bands and four conduction bands in *BSE* calculation, from which we obtain the dispersion of eight exciton bands, naturally embracing the interlayer exciton and the intralayer A/B exciton states of bilayer 2*H*-MoS_2.

To get the mode- and momentum-resolved coupling strength for the exciton-phonon coupling, we start from a perturbation theory-based framework[18,19], where the total energy of an electron-phonon coupled system can be described as a summation of its ground state energy and a linear expansion with respect to the lattice displacement

$$E\{\tau_{\kappa\alpha i}, \psi\} = E\{\tau^0_{\kappa\alpha i}, \psi^0_{n\mathrm{k}}\} + \frac{1}{2}C_{\kappa_1\alpha i,\kappa_2\beta j}\tau_{\kappa_1\alpha i}\tau_{\kappa_2\beta j} + \int \psi^*\left[H^0_{KS} + \frac{\partial V^0_{KS}}{\partial\tau_{\kappa_1\alpha i}}\tau_{\kappa_1\alpha i}\right]\psi \quad (4)$$

Here ψ is the electron's wave function, $\tau_{\kappa\alpha i}$ the atomic displacement with $\kappa\alpha i$ denoting the Cartesian coordinate α of atom κ in the ith unit cell. H^0_{KS}, $\frac{\partial V^0_{KS}}{\partial\tau_{\kappa_1\alpha i}}$, and $\psi^0_{n\mathrm{k}}$ are the ground state Kohn-Sham (KS) Hamiltonian, the variation of the KS potential, and the wave function with band index n and wave vector k, respectively. $C_{\kappa_1\alpha i,\kappa_2\beta j}$ represents the force constant matrix. The total energy for an exciton-phonon coupled system can be expressed by replacing the KS Hamiltonian with the many-body *BSE* Hamiltonian and the electronic wave function by its exciton counterpart

$$E\{\tau_{\kappa\alpha i}, \psi_{ex}\} = E\{\tau^0_{\kappa\alpha i}, \psi^0_{ex}\} + \frac{1}{2}C_{\kappa_1\alpha i,\kappa_2\beta j}\tau_{\kappa_1\alpha i}\tau_{\kappa_2\beta j} + \int \psi^*_{ex}\left[H_{BSE} + \frac{\partial H_{BSE}}{\partial\tau_{\kappa_1\alpha i}}\tau_{\kappa_1\alpha i}\right]\psi_{ex}, \quad (5)$$

where ψ^0_{ex} denotes the exciton empty state. The *BSE* Hamiltonian can be written as $H^{BSE}_{vc,v'c'} = (\varepsilon_c - \varepsilon_v)\delta_{cc'}\delta_{vv'} + (2V_{vc,v'c'} - W_{vc,v'c'})$, with W and V representing the direct electron-electron attraction and the exchange term, respectively. $c(c')$and $v(v')$are the conduction and valence band indices.

Applying the principles of energy minimization and exciton number conservation to Eq. 5, we reach the following self-consistent eigen equations[20,21]:

$$\frac{2}{N_p} B_{\mathbf{q}\mu} G_{nmv}^{ex-ph}(\mathbf{Q},\mathbf{q}) A_{m\mathbf{Q+q}} = \left(\varepsilon_{n\mathbf{Q}} - \varepsilon\right) A_{n\mathbf{Q}},$$

$$B_{\mathbf{q}\mu} = \frac{1}{N_p} A^{*}_{m\mathbf{Q+q}} \frac{G_{nmv}^{ex-ph}(\mathbf{Q},\mathbf{q})}{\hbar\omega_{\mathbf{q}\mu}} A_{n\mathbf{Q}}. \qquad (6)$$

Here N_p is the number of unit cells in the supercell. $A_{n\mathbf{Q}}$ denotes the wave function in the exciton basis, with $B_{\mathbf{q}\nu}$ the lattice wave function in the phonon eigenmode basis. The exciton-phonon coupling matrix $G_{nmv}^{ex-ph}(\mathbf{Q},\mathbf{q})$ is defined by the differential of the *BSE* Hamiltonian with respect to lattice displacement along a phonon normal mode. Assuming constant static screening function, one can estimate the G_{nmv}^{ex-p} $(\mathbf{Q},\mathbf{q})$ using the exciton wave function in the electron-hole pair basis $E^{n\mathbf{Q}}_{v\mathbf{k},c\mathbf{k+Q}}$ and electron-phonon coupling matrix g^{el-ph} by

$$G_{nm\mu}^{ex-ph}(\mathbf{Q},\mathbf{q}) = E^{m\mathbf{Q+q}*}_{v\mathbf{k},c\mathbf{k+Q+q}} E^{n\mathbf{Q}}_{v\mathbf{k},c'\mathbf{k+Q}} g^{el-ph}_{cc'\mu}(\mathbf{k+Q},\mathbf{q}) - E^{m\mathbf{Q+q}*}_{v\mathbf{k-q},c\mathbf{k+Q}} E^{n\mathbf{Q}}_{v'\mathbf{k},c\mathbf{k+Q}} g^{el-ph}_{vv'\mu}(\mathbf{k-q},\mathbf{q}), \qquad (7)$$

where m and n denote the exciton band indices and $\mathbf{Q}$ is the exciton center-of-mass momentum.

Supplementary References:

1 Gong, Z. *et al.* Magnetoelectric effects and valley-controlled spin quantum gates in transition metal dichalcogenide bilayers. *Nat. Commun.* **4**, 2053 (2013).

2 Du, L. *et al.* Strongly distinct electrical response between circular and valley polarization in bilayer transition metal dichalcogenides. *Phys. Rev. B* **99**, 195415 (2019).

3 Zhao, Y. *et al.* Interlayer exciton complexes in bilayer MoS_2. *Phys. Rev. B* **105**, L041411 (2022).

4 Johnson, B. B. & Peticolas, W. L. The Resonant Raman Effect. *Ann. Rev. Phys. Chem.* **27**, 465-521 (1976).

5 del Corro, E. *et al.* Excited Excitonic States in 1L, 2L, 3L, and Bulk WSe_2 Observed by Resonant Raman Spectroscopy. *ACS Nano* **8**, 9629-9635 (2014).

6 Carvalho, B. R., Malard, L. M., Alves, J. M., Fantini, C. & Pimenta, M. A. Symmetry-Dependent Exciton-Phonon Coupling in 2D and Bulk MoS_2 Observed by Resonance Raman Scattering. *Phys. Rev. Lett.* **114**, 136403 (2015).

7 del Corro, E. *et al.* Atypical Exciton–Phonon Interactions in WS_2 and WSe_2 Monolayers Revealed by Resonance Raman Spectroscopy. *Nano Lett.* **16**, 2363-2368 (2016).

8 Jin, C. *et al.* Interlayer electron–phonon coupling in WSe_2/hBN heterostructures. *Nat. Phys.* **13**, 127-131 (2017).

9 Du, L. *et al.* Strong and tunable interlayer coupling of infrared-active phonons to excitons in van der Waals heterostructures. *Phys. Rev. B* **99**, 205410 (2019).

10 Du, L. *et al.* Robust circular polarization of indirect Q-K transitions in bilayer 3R WS_2. *Physical Review B* **100**, 161404 (2019).

11 Fano, U. Effects of Configuration Interaction on Intensities and Phase Shifts. *Phys. Rev.* **124**, 1866-1878 (1961).

12 Miroshnichenko, A. E., Flach, S. & Kivshar, Y. S. Fano resonances in nanoscale structures. *Rev. Mod. Phys.* **82**, 2257-2298 (2010).

13 Tan, P. H. *et al.* The shear mode of multilayer graphene. *Nat. Mater.* **11**, 294-300 (2012).

14 Tan, Q.-H. *et al.* Observation of forbidden phonons, Fano resonance and dark excitons by resonance Raman scattering in few-layer WS_2. *2D Mater.* **4**, 031007 (2017).

15 Marini, A., Hogan, C., Grüning, M. & Varsano, D. yambo: An ab initio tool for excited state calculations. *Comput. Phys. Commun.* **180**, 1392-1403 (2009).

16 Deslippe, J. *et al.* BerkeleyGW: A massively parallel computer package for the calculation of the quasiparticle and optical properties of materials and nanostructures. *Comput. Phys. Commun.* **183**, 1269-1289 (2012).

17 Sangalli, D. *et al.* Many-body perturbation theory calculations using the yambo code. *J. Phys.: Condens. Matter* **31**, 325902 (2019).

18 Sio, W. H., Verdi, C., Poncé, S. & Giustino, F. Polarons from First Principles, without Supercells. *Phys. Rev. Lett.* **122**, 246403 (2019).

19 Sio, W. H., Verdi, C., Poncé, S. & Giustino, F. Ab initio theory of polarons: Formalism and applications. *Phys. Rev. B* **99**, 235139 (2019).

20 Antonius, G. & Louie, S. G. Theory of exciton-phonon coupling. *Phys. Rev. B* **105**, 085111 (2022).

21 Chen, H.-Y., Sangalli, D. & Bernardi, M. Exciton-Phonon Interaction and Relaxation Times from First Principles. *Phys. Rev. Lett.* **125**, 107401 (2020).
